# Factors associated with non-use of antenatal iron and folic acid supplements among Pakistani women: a cross sectional household survey

**DOI:** 10.1186/1471-2393-14-305

**Published:** 2014-09-04

**Authors:** Yasir Bin Nisar, Michael J Dibley, Ali Mohammad Mir

**Affiliations:** Sydney School of Public Health, The University of Sydney, Sydney, NSW 2006 Australia; Population Council, Islamabad, Pakistan

**Keywords:** Prevalence, Pregnancy, Iron and folic acid supplements, Socio-demographic factors, Antenatal care

## Abstract

**Background:**

World Health Organization recommends a standard daily oral dose of iron and folic acid (IFA) supplements throughout pregnancy to begin as early as possible. The aim of the present study was to determine the prevalence of use of antenatal IFA supplements, and the socio-demographic factors associated with the non-use of antenatal IFA supplements from 14 selected districts in Pakistan.

**Methods:**

Data was derived from a cross sectional household survey conducted in 14 project districts across Pakistan. Trained female field workers conducted interviews with married women of reproductive age from December 2011 to March 2012. Women with the most recent live births in the preceding five years of the survey were selected for this study. Data was analysed by using STATA 13 and adjusted for the cluster sampling design. Multivariate logistic regression models were constructed to identify the independent factors associated with the non-use of antenatal IFA supplements.

**Results:**

Of 6,266 women interviewed, 2,400 (38.3%, 95% CI, 36.6%, 40.1%) reported taking IFA supplements during their last pregnancy. Among IFA users, the most common source of supplements was doctors (49.4%) followed by community health workers (40.3%). The mean (±SE) number of supplements used was 76.9 (±51.6), and the mean (±SE) month of pregnancy at initiation of supplementation was 5.3 (±1.7) months. Socio-demographic factors significantly associated with the non-use of antenatal IFA supplements were living in Dera Ghazi Khan district (AdjOR: 1.72), maternal age 45 years and above (AdjOR: 1.97), no maternal education (AdjOR: 2.36), no paternal education (AdjOR: 1.58), belonging to the lowest household wealth index quartile (AdjOR: 1.47), and no use of antenatal care (ANC) services (AdjOR: 13.39).

**Conclusions:**

The coverage of antenatal IFA supplements is very low in the surveyed districts of Pakistan, and the lack of parental education, older aged women, belonging to poorest households, residence in Dera Ghazi Khan district and no use of ANC services were all significantly associated with non-use of these supplements. These findings highlight the urgent need to develop interventions targeting all pregnant women by improving ANC coverage to increase the use of antenatal IFA supplements in Pakistan.

**Electronic supplementary material:**

The online version of this article (doi:10.1186/1471-2393-14-305) contains supplementary material, which is available to authorized users.

## Background

Globally, 42% of pregnant women are anaemic [1] and approximately half of this burden is assumed to be due to iron deficiency [2]. In Pakistan, 51% of pregnant women are anaemic [3]. Several studies have reported associations of anaemia during pregnancy with the risk of maternal mortality [4] and poor pregnancy outcomes in terms of low birth weight [5–7], and prematurity [5, 7, 8], which is the leading cause of neonatal mortality in developing countries [9]. A community based trial from China found a 47% reduction in neonatal mortality in women who received iron and folic acid (IFA) supplements compared with folic acid alone [10]. Therefore, to reduce the risk of maternal anaemia, iron deficiency and poor pregnancy outcomes, the World Health Organization (WHO) guidelines recommend a standard daily oral dose of 30-60 mg iron and 400 μg folic acid supplements throughout pregnancy, to begin as early as possible as a part of antenatal care (ANC) programs. In addition, where the prevalence of anaemia in pregnancy is over 40%, a daily dose of 60 mg of elemental iron is preferred over a lower dose of 30 mg [11].

In Pakistan, IFA supplements are distributed by the maternal and child health services through the existing primary healthcare system including community health workers [Lady Health Workers Program] and health facilities. However, the findings of the latest Pakistan Demographic and Health Survey (DHS) 2012-13 revealed that only 45% of pregnant women consumed antenatal IFA supplements in their most recent pregnancy, while it was much lower (39%) among rural pregnant women [12]. Compared with other South Asian countries [13–16] the prevalence of use of antenatal IFA supplements in Pakistan is the lowest [12]. This indicates that antenatal IFA supplementation programs in Pakistan are being poorly implemented, especially in rural areas. Several factors have been documented, which play important roles in the implementation of IFA supplementation programs. Some of these factors are related to the health system directly, such as, inadequate coverage of populations in need of services, lack of political commitment and financial support, deficiencies in supply and distribution of the supplements at health centres and/or with community health workers, inadequate training of health workers, and the presentation and characteristics of the supplements. While other factors are associated with the clients, such as, inadequate information about benefits of supplements, their cultural and health beliefs, and undesirable side effects associated with intake of IFA supplements [17].

Socio-demographic and healthcare utilization factors of pregnant women also play vital role in the uptake of antenatal IFA supplements. Previous studies have found several factors associated with the use of antenatal IFA supplements. These are: the age of the woman [18–20], her educational status [20–22], her working status [18], her smoking status [19, 22], her alcohol intake [19], the socio-economic status of her family [19], her parity [18, 19, 21, 23–25], the number of IFA supplements received [26], her use of ANC services [18, 24, 26–28], her place of residence/ region [18, 22], and her partner’s occupation [18].

As the coverage of antenatal IFA supplements is low in Pakistan, there is a need to determine the relationship between socio-demographic factors and the use of these supplements. There is no published literature available on this topic from Pakistan. The findings of the current study will help stakeholders from government and non-governmental organizations (NGOs) to formulate strategies and interventions for efficient distribution of the antenatal IFA supplements to target populations through the existing programs. The aim of the current study was to describe the use of antenatal IFA supplements and to identify socio-demographic factors of pregnant women associated with non-use of antenatal IFA supplements in 14 selected districts of Pakistan.

## Methods

### Data source

Data used for the current study were derived from the end line household survey of Family Advancement for Life and Health (FALAH) project. The FALAH was a district-level, five-year (2007-2012) project funded by the United States Agency for International Development (USAID) to improve the survival and health of women and children in Pakistan and the well-being of families, communities and the country through increased demand and utilization of birth spacing and quality family planning services. Initially, the project was conducted in 20 districts with poorer reproductive health indicators across Pakistan. However, from year 4 onwards the project activities were restricted to 15 districts.

### FALAH end line survey

**Figure 1:**
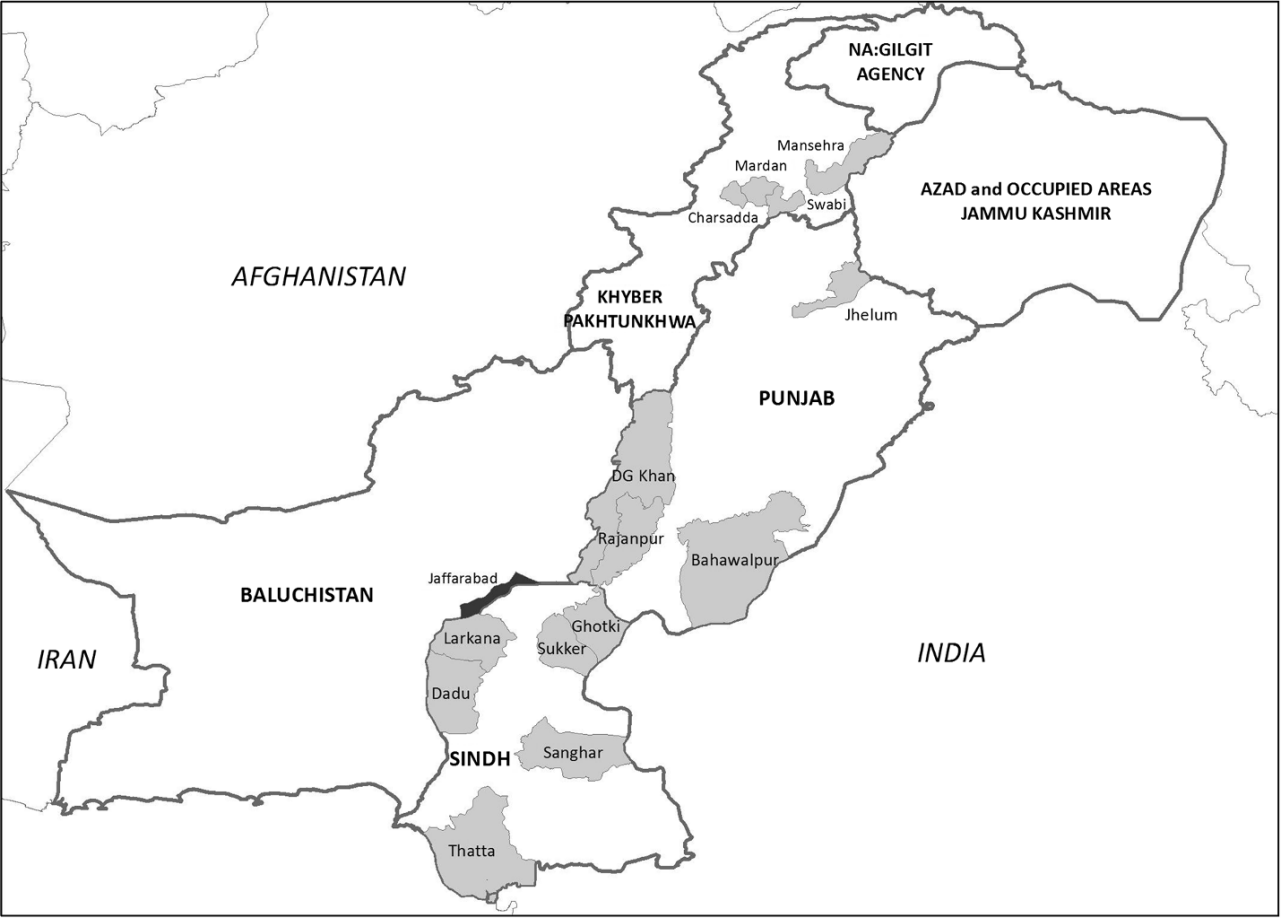
**Map of Pakistan showing FALAH project districts.** Legends: Gray color: Surveyed district. Black color: Survey cancelled. Gray color lines: Province boundary. DG Khan: Dera Ghazi Khan.

The end line survey was conducted in 14 districts, 6 in Sindh, and 4 each in Punjab and Khyber Pakhtunkhwa provinces (Figure 1). The survey was not conducted in Jaffarabad district in Balochistan province due to the poor law and order situation. The survey was conducted to determine the level of awareness about birth spacing and its impact on maternal and child health; trends in contraceptive prevalence rate and the unmet need for contraception; reasons for discontinuation; impact of specific interventions by the project; and to record the complete reproductive history, including ANC services and IFA supplements intake, during the last pregnancy within five years prior to the survey. Field workers were hired and trained in interview techniques and the use of the survey questionnaires before the commencement of the data collection. For each district, a team of four female interviewers, a male interviewer and a male supervisor was selected to conduct the interviews with currently married women of reproductive age (15-49 years).

A systematic two-stage stratified random cluster sampling design was used to select a representative sample of each district. The sample was designed to provide reliable estimates for a variety of reproductive health variables at district level. The universe consisted of all urban and rural households in each district. In urban areas, the clusters were selected from a list of enumeration blocks maintained by the Federal Bureau of Statistics and considered as the primary sampling unit (PSU). Each block consisted of approximately 250-300 households. For the rural sample, the lists of villages in each district enumerated in the 1998 population census were considered as the PSU. First, a total of 40 clusters per district were selected, with probability proportional to size and stratified by urban and rural residence. Afterwards, a fixed number of 15 households (secondary sampling units) within each sample PSUs were selected by systematic random sampling technique. Within each household, all married women of reproductive age (15-49 years) were interviewed. Data collection was started in December 2011 and was completed in March 2012. A total of 8,490 households and 12,402 women of reproductive age were interviewed in the survey. Women with the most recent live birth five years preceding the survey were selected for the current analysis.

An informed written consent was obtained from all the respondents before the commencement of interview. The FALAH project activities were approved by the Institutional Review Board of the Population Council and the Ministry of Health, Pakistan. In addition, the ethics approval of the current study protocol was also obtained from the Human Research Ethics Committee of The University of Sydney, Australia. In reporting this study, guidelines from Strengthening the Reporting of Observational Studies in Epidemiology (STROBE) group [29] were followed (Additional file 1).

### Data management and monitoring

Data processing was started in the field with checking of the completed questionnaires by the team supervisors. Editing instructions were provided to the supervisors and emphasis was placed on the importance of completing each questionnaire, correctly identifying each eligible respondent, and the completeness of the household composition. Consistency checks of the data were performed, and the data were edited as appropriate. To ensure the quality of the data, regular monitoring and validation were also carried out throughout the data collection phase.

### Description of variables

#### Use of antenatal IFA supplements

The information about the use of antenatal IFA supplements was obtained from woman who had a live birth within five years preceding the survey, by asking the following questions in the survey: “During this pregnancy, were you given or did you buy any iron-folic acid tablets?” and “During the whole pregnancy, for how many days did you take the tablets?” A mother was categorised as using any antenatal IFA supplements if she took supplements for at least a day during her pregnancy. Each woman who reported taking supplements was further asked about the source, the number of supplements used, and the month of her pregnancy at the start of supplementation. Use of antenatal IFA supplements was treated as a binary outcome (no use or ever used in pregnancy) in all analyses.

### Socio-demographic and healthcare utilization

Socio-demographic and healthcare utilization factors used in the current study included woman’s age in years (15 to 24 years, 25 to 34 years, 35 to 44 years and 45 years and more), her working status, her and her husband’s educational status categorised as: above secondary (above grade 8 of schooling), at least some secondary school (grade 6-8 of schooling), at least some primary school (grade 1-5 of schooling), and no education, outcome of her last live birth within five years preceding the survey (singleton or multiple), duration since her last birth within 5 years preceding the survey (within 1 year, 1-2 years and 3 and more years), her number of live births within 5 years preceding the survey (one, two, and 3 or more), place of residence (urban/ rural), districts, lady health workers program areas, ANC services by type of providers and household wealth index. In Pakistan, ANC services are provided through static health facilities in both rural and urban areas while in many rural areas lady health workers program provide ANC services. The lady health workers program was launched in 1994 to improve maternal and child health in low-income communities in Pakistan. One lady health worker, a female community health worker, is responsible for approximately 1,000 residents, or 150 homes, and she visits 5 to 7 of these homes every day [30]. They are responsible for providing health education and IFA supplements during ANC visits to all pregnant women from the second trimester of pregnancy in their catchment area.

The household wealth index was constructed from an inventory of household assets and facilities. The weighting values for the indicator variables were assigned using principal components analysis [31]. This index gives each household a score on each of the following variables: source of drinking water; toilet facilities; material of floor; availability of electricity; ownership of a radio; ownership of a television set; ownership of a refrigerator; and means of transportation. The household wealth index was the sum of the weighted scores for each item. For analysis the household wealth index scores were ranked and divided into quartiles [32].

### Statistical analysis

Data analysis was conducted by using STATA 13 (Stata- Corp, College Station, TX, USA) with ‘svy’ commands to allow for adjustments for the cluster sampling design used in the survey. We conducted frequency tabulations to describe (a) the study population; (b) the use of antenatal IFA supplements; and (c) the source, the number of supplements used, and the month of pregnancy at the start of supplementation. Afterwards, we compared the socio-demographic characteristics of the respondents with the number of supplements used (none, <60, 60-89 and ≥90) using χ-square test for trend analysis. Then, we compared the socio-demographic characteristics between the supplement users and the non-users by using χ-square test. Unadjusted odds ratio (OR), 95% confidence intervals (CI) and p-values (p) were reported. Finally, we constructed multivariate logistic regression models by the backwards elimination method to identify the independent predictors of the non-use of IFA supplements among women. All variables, which were statistically significant (p ≤ 0.10) in the univariate analysis, were included in the regression models. Adjusted OR, 95% CI and p were reported. The level of significance was considered at 5%.

## Results

### Socio-demographic characteristics of respondents

There were 6,266 women interviewed and their socio-demographic characteristics are shown in Table 1. The sample for women interviewed varied from 668 (10.7%) in Bahawalpur district, to 239 (3.8%) in district Jhelum. The vast majority of these women were living in rural areas (81.2%). Seventy-two per cent of respondents were living in lady health worker program areas. Fifty-six per cent of respondents had 2 or more live births within the last 5 years prior to the survey. One third of the respondents did not use ANC services during their last pregnancy.

**Table 1:** **Baseline characteristics of all women with the most recent live births within 5 years prior to the survey in 14 districts of Pakistan (n = 6,266)**

Variable	Number	%
**District**		
Jhelum	239	3.8
Swabi	312	5.0
Charsadda	331	5.3
Sukkur	348	5.6
Ghotki	370	5.9
Mansehra	381	6.1
Thatta	382	6.1
Rajanpur	459	7.3
Mardan	492	7.8
Sanghar	522	8.3
Dera Ghazi Khan	572	9.1
Dadu	572	9.1
Larkana	619	9.9
Bahawalpur	668	10.7
**Place of residence**		
Urban	1,178	18.8
Rural	5,089	81.2
**Lady health worker program area**		
Yes	4,538	72.4
No	1,728	27.6
**Age of respondents**		
Mean (SE)	29.6 (±6.5)	
Median (IQR‡)	29.0 (25.0 - 34.0)	
**Age categories of respondents**		
15 to 24 years	1,460	23.3
25 to 34 years	3,293	52.6
35 to 44 years	1,392	22.2
45 years and more	121	1.9
**Educational status of respondents**		
Above secondary	354	5.6
At least some secondary school	798	12.7
At least some primary school	910	14.5
No education	4,205	67.1
**Working status of respondents**		
Not working	4,326	69.0
Working	1,930	30.8
Missing	10	0.2
**Educational status of respondents’ husband**		
Above secondary	1,098	17.5
At least some secondary school	1,834	29.3
At least some primary school	988	15.8
No education	2,292	36.6
Missing	54	0.9
**Husband's occupation**		
Agriculture/Livestock/Poultry	1,297	20.7
Petty trader	627	10.0
Labourer (Daily wages)	2,258	36.0
Government service	675	10.8
Private service	392	6.3
Own business	247	3.9
Abroad	220	3.5
Unemployed	321	5.1
Others	195	3.1
Missing	34	0.5
**Household wealth index**		
Highest	1,239	19.8
Medium high	1,597	25.5
Medium low	1,708	27.3
Lowest	1,722	27.5
**Outcome of last live birth within 5 years preceding the survey**		
Singleton	6,180	98.6
Multiple	86	1.4
**Duration since last live birth within 5 years preceding the survey**		
Within 1 year	2,014	32.1
1 to 2 years	2,953	47.1
3 and more years	1,299	20.7
**Number of live births within 5 years preceding the survey**		
One	2,746	43.8
Two	2,578	41.1
Three and more	943	15.0
**Antenatal services by type of providers**		
Health professionals	3,931	62.7
Untrained providers	109	1.7
No services	2,094	33.4
Missing	132	2.1

‡Inter-quartile range.

### Prevalence, source, number of supplements used and start of supplementation

There were 2,400 women (38.3%, 95% CI 36.6%, 40.1%) who reported taking antenatal IFA supplements at some stage during their last pregnancy. The most common source of the antenatal IFA supplements was doctors followed by lady health workers (community health workers). Twenty-nine per cent of the women using IFA consumed 120 and more supplements. The mean (±SE) number of the supplements used was 76.9 (±51.6) with a median of 60 supplements. Supplementation was initiated late, on average in the fifth month of pregnancy, and only 5% initiated the supplements during their first trimester of the pregnancy (Table 2).

**Table 2:** **Sources, number of supplements used and start of IFA supplements of women who had the most recent live births within 5 years prior to the survey in 14 surveyed districts of Pakistan (n = 2,400)**

Variable	Number	%
**Sources of IFA supplements**		
Doctor	1,185	49.4
Lady health workers	967	40.3
Nurse/Lady health visitor/ dispenser	121	5.0
Traditional birth attendant/Dai/ midwife	15	0.6
Myself/Husband/Friend	50	2.1
Missing	62	2.6
**Number of IFA supplements used**		
Mean (SE)	76.9 (±51.6)	
Median (IQ Range‡)	60.0 (30.0 - 120.0)	
**Categories of number of IFA supplements used**		
Less than 60	832	34.7
60 to 89	448	18.7
90 to 119	370	15.4
120 and above	688	28.7
Missing	62	2.6
**Month of pregnancy at start of IFA supplements**		
Mean (SE)	5.3 (±1.7)	
Median (IQ Range)	5.0 (4.0 - 7.0)	
Gestation at start of IFA supplements		
First trimester	125	5.2
Second trimester	1,602	66.8
Last trimester	611	25.5
Missing	62	2.6

‡ Inter-quartile range.

### Socio-demographic characteristics of respondents by number of supplements used

Table 3 presents a comparison of the socio-demographic characteristics of the respondents by the number of IFA supplements consumed. Twenty-four per cent of women living in urban areas consumed 90 or more supplements. Nineteen per cent of women living in the lady health workers program areas consumed 90 or more supplements, but only 12% of women in non-lady health workers program areas. Among women who reported to have had ANC services by health professionals, 25% of them consumed 90 or more supplements during their last pregnancy but only 2.8% amongst those women who reported no use of any ANC services.

**Table 3:** **Basic characteristics of women by number of IFA supplements consumed during the most recent pregnancy within 5 years prior to the survey in 14 surveyed districts in Pakistan (n = 6,266)**

Variables	Number of IFA supplements consumed										p
	None		Less than 60		60 to 89		90 and above		Missing		
	n	%	n	%	n	%	n	%	n	%	
District											0.0001
Jhelum	102	42.5	56	23.4	22	9.3	55	22.9	4	1.9	
Charsadda	165	49.8	50	15.1	36	10.8	79	23.9	1	0.3	
Sukkur	188	53.9	65	18.6	22	6.4	69	19.9	4	1.3	
Swabi	171	54.7	37	11.7	24	7.8	80	25.8	0	0.0	
Sanghar	292	55.9	84	16.1	42	8.1	101	19.4	3	0.5	
Thatta	214	56.1	80	20.9	31	8.1	48	12.6	9	2.4	
Mansehra	226	59.5	37	9.6	28	7.4	88	23.2	1	0.3	
Mardan	292	59.5	44	8.9	52	10.7	103	21.0	0	0.0	
Larkana	382	61.8	110	17.7	38	6.1	85	13.8	4	0.6	
Dadu	360	62.9	49	8.6	39	6.8	114	20.0	10	1.8	
Ghotki	246	66.5	56	15.2	23	6.1	41	11.0	4	1.2	
Bahawalpur	457	68.4	63	9.5	44	6.6	97	14.6	6	0.9	
Rajanpur	332	72.5	40	8.8	23	5.0	57	12.4	7	1.4	
Dera Ghazi Khan	440	76.9	62	10.9	24	4.1	39	6.7	7	1.3	
**Place of residence**											0.0001
Urban	595	50.5	177	15.1	104	8.8	280	23.8	22	1.9	
Rural	3,272	64.3	655	12.9	344	6.8	777	15.3	40	0.8	
**Lady health worker program area**											0.0001
Yes	2,670	58.8	624	13.8	338	7.4	856	18.9	50	1.1	
No	1,197	69.3	208	12.1	111	6.4	201	11.6	11	0.7	
**Age of respondents**											0.0001
15 to 24 years	849	58.1	239	16.3	112	7.7	252	17.3	9	0.60	
25 to 34 years	1,996	60.6	432	13.1	241	7.3	587	17.8	37	1.1	
34 to 44 years	923	66.3	154	11.0	93	6.7	207	14.9	15	1.1	
45 years and more	98	81.4	8	6.4	3	2.3	11	9	1	0.9	
**Educational status of respondents**											0.0001
Above secondary	101	28.6	45	12.8	49	13.9	150	42.4	8	2.4	
At least some secondary school	324	40.6	130	16.3	83	10.4	245	30.7	16	2	
At least some primary school	486	53.4	145	15.9	68	7.5	197	21.6	15	1.6	
No education	2,956	70.3	512	12.2	248	5.9	465	11.1	23	0.5	
**Working status of respondents**											0.0001
Not working	2,516	58.2	608	14	333	7.7	821	19	49	1.1	
Working	1,345	69.7	223	11.6	114	5.9	236	12.2	13	0.7	
Missing	6	59.6	2	18.6	2	21.8	0	0	0	0	
**Educational status of respondents’ husband**											0.0001
Above secondary	454	41.3	157	14.3	123	11.2	343	31.3	21	1.9	
At least some secondary school	1,050	57.2	262	14.3	144	7.9	359	19.6	19	1	
At least some primary school	647	65.4	138	13.9	69	6.9	126	12.8	9	0.9	
No education	1,684	73.5	267	11.7	110	4.8	218	9.5	12	0.5	
Missing	32	59.5	9	16.2	3	5.7	10	18.6	0	0	
**Household wealth index**											0.0001
Highest	542	43.8	178	14.3	137	11	355	28.7	27	2.2	
Medium high	865	54.2	241	15.1	120	7.5	360	22.5	11	0.7	
Medium low	1,172	68.6	219	12.8	103	6	207	12.1	8	0.5	
Lowest	1,287	74.7	195	11.3	89	5.2	136	7.9	16	0.9	
**Outcome of last live birth within 5 years preceding the survey**											0.092
Singleton	3,814	61.7	827	13.4	444	7.2	1,033	16.7	61	0.99	
Multiple	52	60.4	5	6.1	4	5.1	24	27.4	1	1.04	
**Duration since last birth within 5 years preceding the survey**											0.0003
Within 1 year	1,176	58.4	313	15.6	163	8.1	354	17.6	8	0.4	
1 to 2 years	1,833	62.1	383	13	204	6.9	498	16.9	35	1.2	
3 and more years	858	66	136	10.5	82	6.3	205	15.8	19	1.5	
**Number of live births within 5 years preceding the survey**											0.038
One	1,702	62	320	11.7	207	7.55	488	17.8	28.3	1	
Two	1,570	60.9	359	13.9	187	7.23	438	17	25.1	1	
Three and more	595	63.1	154	16.3	55	5.82	131	13.9	8.4	0.9	
**Antenatal services by type of providers**											0.0001
Health professionals	1,739	44.2	753	19.2	401	10.2	982	25	56	1.4	
Untrained providers	62	56.7	19	17.7	12	11	14	13	2	1.6	
No services	1,942	92.7	59	2.8	32	1.5	58	2.8	3	0.1	
Missing	124	94.1	1	0.8	3	2.4	2	1.9	1	0.8	

Chi-square for trend was used to obtained p-values for each variable.

### Socio-demographic factors associated with the non-use of antenatal IFA supplements

The multivariate analysis, as presented in Table 4, found that women who had most recent live birth within 5 years prior to the survey, living in Dera Ghazi Khan district (AdjOR: 1.72, p = 0.032), aged 45 years and above (AdjOR: 1.97, p = 0.029), with no education (AdjOR: 2.36, p < 0.0001), with a husband who had no education (AdjOR: 1.58, p < 0.0001), belonging to the lowest household wealth index quartile (AdjOR: 1.47, p = 0.006), and who did not utilize ANC services during their last pregnancy (AdjOR: 13.39, p < 0.0001) were significantly associated with the non-use of antenatal IFA supplements in the surveyed districts of Pakistan. To address the extent of recall bias we compared the prevalence of non-use of IFA supplements (58%, 95% CI 55.6%, 61.1%) reported by women who had delivered within 1 year prior to the survey with the reports from women who had delivered 3 or more years prior to the survey (66%, 95% CI 62.7%, 69.2%) which was statistically significant. Further, we also analysed the risk factors for non-use of IFA supplements in women who had the most recent live birth within 3 years prior to the survey. The factors associated with non-use of IFA supplements in women with the most recent live birth within 3 years prior to survey were identical to those within 5 years prior to the survey (Additional file 2).

**Table 4:** **Risk factors for non-use of iron/folic acid (IFA) supplements during pregnancy in women with the most recent live births within 5 years prior to the surveyed in 14 surveyed districts in Pakistan, findings of univariate and multivariate logistic regression**

Variable	Women who did not use iron supplements		Unadjusted				Adjusted			
	n	%	OR ^1^	95% CI ^2^		p	OR ^1^	95% CI ^2^		p
District										
Jhelum	102	42.5	1.00				1.00			
Charsadda	165	49.8	1.34	0.9	1.99	0.143	0.38	0.23	0.62	<0.0001
Sukkur	188	53.9	1.58	1.02	2.43	0.038	0.77	0.48	1.21	0.252
Swabi	171	54.7	1.63	1.02	2.62	0.043	0.58	0.35	0.95	0.029
Sanghar	292	55.9	1.71	1.18	2.49	0.005	0.47	0.3	0.74	0.001
Thatta	214	56.1	1.72	1.16	2.56	0.007	0.58	0.38	0.91	0.016
Mansehra	226	59.5	1.98	1.31	3.01	0.001	1.19	0.78	1.81	0.431
Mardan	292	59.5	1.98	1.31	2.99	0.001	0.8	0.51	1.25	0.328
Larkana	382	61.8	2.18	1.48	3.22	0.0001	0.94	0.6	1.47	0.792
Dadu	360	62.9	2.29	1.52	3.45	0.0001	0.94	0.61	1.46	0.792
Ghotki	246	66.5	2.68	1.86	3.88	0.0001	1.09	0.71	1.67	0.704
Bahawalpur	457	68.4	2.92	1.92	4.43	0.0001	1.49	0.96	2.34	0.078
Rajanpur	332	72.5	3.55	2.39	5.28	0.0001	1.09	0.7	1.69	0.699
Dera Ghazi Khan	440	76.9	4.51	2.89	7.03	0.0001	1.72	1.05	2.82	0.032
**Place of residence**										
Urban	595	50.5	1.00							
Rural	3,272	64.3	1.77	1.48	2.10	0.0001	NS			
**Lady health worker program area**										
Yes	2,670	58.8	1.00							
No	1,197	69.3	1.58	1.34	1.85	0.0001	NS			
**Age of respondents**										
15 to 24 years	849	58.1	1.00				1.00			
25 to 34 years	1,996	60.6	1.10	0.95	1.27	0.200	1.07	0.90	1.28	0.409
35 to 44 years	923	66.3	1.37	1.14	1.14	0.001	1.12	0.91	1.39	0.290
45 years and more	98	81.4	3.11	1.84	184	<0.0001	1.97	1.07	3.62	0.029
**Educational status of respondents**										
Above secondary	101	28.6	1.00				1.00			
At least some secondary school	324	40.6	1.71	1.25	2.33	0.001	1.27	0.89	1.82	0.197
At least some primary school	486	53.4	2.86	2.08	3.92	0.0001	1.70	1.16	2.49	0.006
No education	2,956	70.3	5.91	4.48	7.79	0.0001	2.36	1.65	3.37	<0.0001
**Working status of respondents**										
Not working	2,516	58.2	1.00							
Working	1,345	69.7	1.65	1.43	1.91	0.0001	NS			
**Educational status of respondents’ husband**										
Above secondary	454	41.3	1.00				1.00			
At least some secondary school	1,050	57.2	1.90	1.61	2.25	0.0001	1.32	1.06	1.64	0.014
At least some primary school	647	65.4	2.69	2.23	3.24	0.0001	1.34	1.04	1.72	0.023
No education	1,684	73.5	3.93	3.32	4.66	0.0001	1.58	1.27	1.97	<0.0001
**Household wealth index**										
Highest	542	43.8	1.00				1.00			
Medium high	865	54.2	1.52	1.25	1.84	0.0001	1.02	0.81	1.27	0.890
Medium low	1,172	68.6	2.81	2.33	3.39	0.0001	1.34	1.05	1.71	0.017
Lowest	1,287	74.7	3.80	3.14	4.60	0.0001	1.47	1.11	1.93	0.006
**Outcome of last live birth within 5 years preceding the survey**										
Singleton	3,814	61.7	1.00							
Twins	52	60.4	0.94	0.56	1.59	0.831	NS			
**Duration since last birth within 5 years preceding the survey**										
Within 1 year	1,176	58.4	1.00							
1 to 2 years	1,833	62.1	1.17	1.01	1.35	0.035	NS			
3 and more years	858	66.0	1.38	1.16	1.66	<0.0001				
**Number of live births within 5 years preceding the survey**										
One	1,702	62.0	1.00							
Two	1,570	60.9	0.95	0.83	1.09	0.505	NS			
Three and more	595	63.1	1.05	0.87	1.27	0.622				
**Antenatal services by type of providers**										
Health professionals	1,739	44.2	1.00				1.00			
Untrained providers	62	56.7	1.65	1.05	2.59	0.029	1.44	0.91	2.27	0.130
No services	1,942	92.7	16.07	12.99	19.88	<0.0001	13.39	10.70	16.75	<0.0001

147 missing values were excluded from the analysis.

Unadjusted and adjusted odds ratio with 95% confidence intervals were obtained using logistic regression analysis.

^1^OR: Odds Ratio.

^2^CI: Confidence Interval.

## Discussion

The prevalence of use of antenatal IFA supplements was low with only 38% of women reporting consumption of antenatal IFA during their last pregnancy. Among the supplement users, more than one third of the women took less than 60 supplements during their pregnancy. A substantial majority of women initiated the supplements in their second trimester of pregnancy, with an average initiation in the fifth month of pregnancy. About two fifths of the women received supplements from community health workers. The non-use of antenatal IFA supplements was associated with women living in Dera Ghazi Khan district, aged 45 years and above, with women and/or their husbands having no education, belonging to the lowest household wealth index group, and who did not receive ANC services. The low prevalence of the use of antenatal IFA supplements and factors associated with non-use of antenatal IFA supplements are important to provide guidance for the development of evidence based approaches directed at increasing the intake and coverage of antenatal IFA supplementation in Pakistan. Subsequently, this will help in reducing the burden of maternal anaemia, low birth weight babies and prematurity, which is one of the leading causes of neonatal mortality in Pakistan [33]. The latest Pakistan Demographic and Health Survey 2012-13 reported that the 45% of mothers reported use of antenatal IFA supplements in the most recent birth 5 years prior to the survey. Moreover, 39% of rural Pakistani pregnant women reported to use antenatal IFA supplements during their last pregnancy 5 years prior to the survey. The highest percentage of women reported to use IFA supplements during their last pregnancy 5 years prior to the survey was reported from Islamabad region (capital city of Pakistan) while lowest percentage was reported from Balochistan province (17%) [12].

The major strength of our study is that it has a large sample, and was a population based study conducted in 14 selected districts across Pakistan with respondents who were selected through a multistage cluster sampling technique to provide samples representative of each selected district. Moreover, female interviewers were hired and trained to conduct the interviews with the respondents in their local languages. Moreover, we found identical risk factors for non-use of IFA supplements during pregnancy in women with the most recent live births either within 5 years prior to the survey or within 3 years of the survey which showed that there was a minimum to no recall bias.

A limitation of this study is the nature of the temporal relationship between socio-demographic and healthcare utilization factors and the status of use of IFA supplements during pregnancy as the information was collected in a cross-sectional survey and both were measured at the same time. This restricts drawing conclusions about causality of the factors examined for the non-use of IFA supplements. Another limitation is the potential for recall bias as we asked about the respondent’s last pregnancy within five years prior to the survey. We observed that women who had more recent births reported significantly lower rates of non-use of IFA supplements.

The results of this study indicated that women who had no education had higher odds of not consuming IFA supplements during their pregnancy. Our study is consistent with surveys from South Asia [12–16, 34] and other studies from other regions, which reported an association between lower educational status and the non-use of supplements [20, 21]. The reason behind this is that the educated women have greater access to information about health in pregnancy, which increases their concern about their health and the health of their future newborns and utility of ANC services, than illiterate women [35]. They also have easier access to health facilities, which provide the IFA supplements.

Age of respondents 45 years and above was identified as one of the independent predictors of the non-use of antenatal IFA supplements in our study. Surveys from Pakistan [12, 34], Bangladesh [16], India [13] and Nepal [14, 15] have also reported a similar finding with a higher proportion of older women not consuming antenatal IFA supplements compared to younger women. However, several studies have found young age of respondents as a risk factor for the non-use of antenatal IFA supplements in their study population [18–20]. Older aged women in developing countries are more likely to have experience with pregnancy and child birth and may not feel the need for IFA supplements as they might not have used them with earlier pregnancies.

Women belonging to the lowest household wealth index group had higher odds of not using antenatal IFA supplements in our sample. Nationally representative surveys from Pakistan [12, 34], Bangladesh [16], India [13] and Nepal [14, 15] have reported higher proportions of women belonging to the lowest household wealth index group did not consume IFA supplements during their last pregnancy. On the other hand, a study from a western country reported higher socioeconomic status as a risk factor for non-use of IFA supplements during pregnancy [19]. In developing countries, women belonging to the poorest families usually do not utilize ANC services due to their limited resources.

Not received any ANC services was significantly associated with the non-use of antenatal IFA supplements in the current study, which is similar to other studies, from Cambodia [26], Tanzania [18] and Philippines [24]. The rate of ANC services provided by health professionals is often low in developing countries, and in Pakistan 73% of women had ANC services from health professionals [12], which could lead to low access to antenatal IFA supplements [27]. Previous studies have reported that women’s limited access to or participation in ANC is one of the major reasons for not to taking supplements in developing countries [28, 36]. Due to limited access to ANC services in developing countries, WHO, therefore, advocates focused ANC comprised of 4 visits [37], rather than the older schedule of monthly visits and to distribute the IFA supplements to all women at each of the 4 visits [38]. Improving the coverage of ANC services provided by health professionals in developing countries will increase use of IFA supplements. However, at the same time there is a need to explore other ways for provision of IFA supplements to pregnant women who cannot utilize ANC services.

The current WHO guidelines recommend initiation of oral daily IFA supplements as early as possible during the pregnancy [11]. However, we found that the supplementation initiation was late on average during the fifth month of pregnancy and 17% of women consumed 90 or more supplements throughout their pregnancy. Early initiation and the total number of supplements consumed during pregnancy have a significant impact on child mortality. In Indonesia a strong dose response has been reported for use of IFA supplements in pregnancy, with the risk of under-five mortality progressively reduced as the total number of IFA supplements consumed increased. Moreover, infants of women, who initiated IFA supplements early in pregnancy, had a greater reduction in the risk of death of children 5 years of age [39]. Hence, early initiation and continued use of supplements is important to reduce neonatal and infant mortality in Pakistan.

The current study findings have implications for the IFA distribution programs in Pakistan as many women receive IFA through lady health worker program. The prevalence of use of IFA supplements in the lady health worker program area was better compared to non-program area in our study. However, there is a need to improve the coverage of IFA supplements in Pakistan. In this context, Nepal has shown substantial progress in antenatal IFA coverage from 23% in 2001 [40] to 80% in 2011 [14] by implementing a district level intervention package. The package consisted of the distribution of supplements through female community health volunteers, improving their counselling skills, conducting awareness campaigns in communities and in the health system, strengthening the logistic system, improving the packaging of the supplements, enhancing ANC services and imparting an effective monitoring system [41]. These interventions could be implemented in Pakistan in collaboration with provincial/district department of health, community health worker program and NGOs. However, to improve the situation in Pakistan further qualitative research is needed to understand the barriers to use of IFA supplements in different settings in the country. Such information will be critical in designing more effective community-based interventions to increase coverage of IFA supplementation in pregnancy. These new approaches will need to be evaluated in community-based trials before up scaling across the country.

## Conclusions

Pakistan has a very low prevalence of use of antenatal IFA supplements and the lack of parental education, older aged women, belonging to the lowest household wealth index, no use of ANC services, and residence in Dera Ghazi Khan district were significantly associated with non-use of these supplements in our sample. These findings highlight that there is a need to develop interventions at the district level to improve the coverage of antenatal IFA supplements in Pakistan. Improved use of antenatal IFA supplements will help reduce anaemia in pregnancy and its impact on risk of maternal mortality and poor pregnancy outcomes such as prematurity and low birth weight, which should subsequently help reduce neonatal and under-five mortality in Pakistan.

## Electronic supplementary material

Additional file 1: **STROBE Statement—Checklist of items that should be included in reports of cross-sectional studies.** (DOC 87 KB)

Additional file 2: Table S1: Risk factors for non-use of iron/folic acid (IFA) supplements during pregnancy in women with the most recent live births within 3 years prior to the surveyed in 14 surveyed districts in Pakistan, findings of univariate and multivariate logistic regression. (DOCX 23 KB)

## Abbreviations

Adj OR: Adjusted odds ratio
ANC: Antenatal care
CI: Confidence interval
DHS: Demographic and Health Survey
FALAH: Family Advancement for Life and Health
IFA: Iron and folic acid
NGOs: Non-governmental organizations
OR: Odds ratio
PSU: Primary sampling unit
SE: Standard error
STROBE: Strengthening the Reporting of Observational Studies in Epidemiology USAID: United States Agency for International Development
WHO: World Health Organization.
