# Supplementary material for: Factors associated with non-use of antenatal iron and folic acid supplements among Pakistani women: a cross sectional household survey
Source: BMC Pregnancy Childbirth. 2014 Sep 4;14:305. doi: 10.1186/1471-2393-14-305 (PMC4162926; doi:10.1186/1471-2393-14-305)
Supplement: Supplementary file 2 — Additional file 2: Table S1: Risk factors for non-use of iron/folic acid (IFA) supplements during pregnancy in women with the most recent live births within 3 years prior to the surveyed in 14 surveyed districts in Pakistan, findings of univariate and multivariate logistic regression. (DOCX 23 KB) [file 12884_2013_1177_MOESM2_ESM.docx]

**Table S1: Risk factors for non-use of iron/folic acid (IFA) supplements during pregnancy in women with the most recent live births within 3 years prior to the surveyed in 14 surveyed districts in Pakistan, findings of univariate and multivariate logistic regression**

|  | **Women who did not use IFA supplements** | | **Unadjusted** | | | | **Adjusted** | | | |
| --- | --- | --- | --- | --- | --- | --- | --- | --- | --- | --- |
| **Variable** | **n** | **%** | **OR^1^** | **95% CI^2^** | | **p** | **OR^1^** | **95% CI^2^** | | **p** |
| **District** |  |  |  |  |  |  |  |  |  |  |
| Jhelum | 70 | 42.0 | 1.00 |  |  |  | 1.00 |  |  |  |
| Swabi | 128 | 53.3 | 1.58 | 0.94 | 2.64 | 0.083 | 0.54 | 0.32 | 0.94 | 0.028 |
| Charsadda | 122 | 47.9 | 1.27 | 0.82 | 1.95 | 0.282 | 0.36 | 0.21 | 0.62 | 0.000 |
| Sukkur | 146 | 54.5 | 1.66 | 1.02 | 2.70 | 0.043 | 0.76 | 0.46 | 1.24 | 0.270 |
| Thatta | 148 | 52.9 | 1.55 | 0.98 | 2.45 | 0.059 | 0.54 | 0.33 | 0.89 | 0.015 |
| Mansehra | 173 | 58.3 | 1.93 | 1.20 | 3.09 | 0.006 | 1.10 | 0.68 | 1.78 | 0.695 |
| Ghotki | 217 | 66.4 | 2.73 | 1.78 | 4.18 | <0.0001 | 1.05 | 0.66 | 1.67 | 0.848 |
| Rajanpur | 271 | 72.4 | 3.62 | 2.34 | 5.61 | <0.0001 | 1.17 | 0.73 | 1.87 | 0.516 |
| Mardan | 211 | 55.1 | 1.70 | 1.07 | 2.70 | 0.025 | 0.65 | 0.40 | 1.07 | 0.092 |
| Sanghar | 238 | 54.4 | 1.64 | 1.08 | 2.51 | 0.021 | 0.50 | 0.30 | 0.82 | 0.006 |
| Dera Gazi Khan | 332 | 75.7 | 4.30 | 2.64 | 6.99 | <0.0001 | 1.68 | 1.00 | 2.85 | 0.050 |
| Dadu | 282 | 61.3 | 2.19 | 1.36 | 3.52 | 0.001 | 0.95 | 0.59 | 1.55 | 0.841 |
| Bahawalpur | 349 | 68.2 | 2.96 | 1.86 | 4.70 | <0.0001 | 1.63 | 1.01 | 2.63 | 0.047 |
| Larkana | 322 | 60.9 | 2.15 | 1.40 | 3.31 | <0.0001 | 0.90 | 0.56 | 1.46 | 0.678 |
| **Place of residence** |  |  |  |  |  |  |  |  |  |  |
| Urban | 450 | 49.2 | 1.00 |  |  |  |  |  |  |  |
| Rural | 2,559 | 63.2 | 1.77 | 1.46 | 2.15 | <0.0001 | NS |  |  |  |
| **Lady health worker program area** |  |  |  |  |  |  |  |  |  |  |
| Yes | 2,054 | 57.4 | 1.00 |  |  |  |  |  |  |  |
| No | 955 | 68.7 | 1.63 | 1.36 | 1.95 | <0.0001 | NS |  |  |  |
| **Age of respondents** |  |  |  |  |  |  |  |  |  |  |
| 15 to 24 years | 759 | 57.3 | 1.00 |  |  |  | 1.00 |  |  |  |
| 25 to 34 years | 1,622 | 60.1 | 1.12 | 0.96 | 1.31 | 0.145 | 1.10 | 0.91 | 1.32 | 0.324 |
| 35 to 44 years | 583 | 64.4 | 1.41 | 1.14 | 1.74 | 0.001 | 1.19 | 0.93 | 1.52 | 0.169 |
| 45 years and more | 46 | 87.0 | 5.00 | 2.20 | 11.36 | <0.0001 | 3.52 | 1.38 | 8.99 | 0.009 |
| **Educational status of respondents** |  |  |  |  |  |  |  |  |  |  |
| Above secondary | 75 | 27.3 | 1.00 |  |  |  | 1.00 |  |  |  |
| At least some secondary school | 257 | 39.5 | 1.74 | 1.20 | 2.53 | 0.004 | 1.36 | 0.90 | 2.05 | 0.142 |
| At least some primary school | 370 | 51.2 | 2.80 | 1.92 | 4.06 | <0.0001 | 1.76 | 1.15 | 2.69 | 0.009 |
| No education | 2,307 | 69.5 | 6.07 | 4.34 | 8.48 | <0.0001 | 2.51 | 1.68 | 3.76 | <0.0001 |
| **Working status of respondents** |  |  |  |  |  |  |  |  |  |  |
| Not working | 1,943 | 56.8 | 1.00 |  |  |  |  |  |  |  |
| Working | 1,061 | 69.0 | 1.70 | 1.45 | 1.99 | <0.0001 | NS |  |  |  |
| **Educational status of respondents’ husband** |  |  |  |  |  |  |  |  |  |  |
| Above secondary | 355 | 40.3 | 1.00 |  |  |  | 1.00 |  |  |  |
| At least some secondary school | 802 | 55.0 | 1.81 | 1.50 | 2.20 | <0.0001 | 1.22 | 0.96 | 1.56 | 0.105 |
| At least some primary school | 518 | 65.9 | 2.87 | 2.31 | 3.57 | <0.0001 | 1.47 | 1.10 | 1.95 | 0.009 |
| No education | 1,309 | 72.6 | 3.93 | 3.22 | 4.79 | <0.0001 | 1.55 | 1.20 | 2.01 | 0.001 |
| **Household wealth index** |  |  |  |  |  |  |  |  |  |  |
| Highest | 413 | 43.0 | 1.00 |  |  |  | 1.00 |  |  |  |
| Medium high | 644 | 52.0 | 1.43 | 1.15 | 1.78 | 0.001 | 0.93 | 0.71 | 1.22 | 0.615 |
| Medium low | 910 | 66.9 | 2.68 | 2.16 | 3.31 | <0.0001 | 1.25 | 0.95 | 1.64 | 0.113 |
| Lowest | 1,042 | 74.0 | 3.77 | 3.03 | 4.68 | <0.0001 | 1.35 | 1.00 | 1.83 | 0.050 |
| **Outcome of last live birth within 3 years preceding the survey** |  |  |  |  |  |  |  |  |  |  |
| Singleton | 2,973 | 60.5 | 1.00 |  |  |  |  |  |  |  |
| Multiple | 36 | 64.4 | 1.18 | 0.63 | 2.19 | 0.603 | NS |  |  |  |
| **Duration since last live birth within 3 years preceding the survey** |  |  |  |  |  |  |  |  |  |  |
| <12 months | 838 | 41.6 | 1.00 |  |  |  |  |  |  |  |
| ≥12 months | 1,120 | 37.9 | 1.17 | 1.01 | 1.35 | 0.035 | NS |  |  |  |
| **Number of live births within 3 years preceding the survey** |  |  |  |  |  |  |  |  |  |  |
| One | 935 | 58.4 | 1.00 |  |  |  |  |  |  |  |
| Two or more | 2,074 | 61.6 | 1.14 | 0.99 | 1.33 | 0.077 | NS |  |  |  |
| **Antenatal services by type of providers** |  |  |  |  |  |  |  |  |  |  |
| Health professionals | 1,430 | 44.4 | 1.00 |  |  |  | 1.00 |  |  |  |
| Untrained providers | 54 | 60.0 | 1.88 | 1.13 | 3.14 | 0.015 | 1.61 | 0.96 | 2.69 | 0.068 |
| No services | 1,515 | 92.5 | 15.38 | 12.23 | 19.34 | <0.0001 | 13.17 | 10.31 | 16.83 | <0.0001 |

54 missing values were excluded from the analysis.

Unadjusted and adjusted odds ratio with 95% confidence intervals were obtained using logistic regression analysis.

^1^OR: Odds Ratio.

^2^CI: Confidence Interval.
